# One Study, Two Paths: The Challenge of Dual-Use Research

**DOI:** 10.1289/ehp.120-a238

**Published:** 2012-06-01

**Authors:** Wendee Holtcamp

**Affiliations:** **Wendee Holtcamp**, based in Houston, Texas, has written for *Nature*, *Scientific American*, *National Wildlife*, and other magazines.


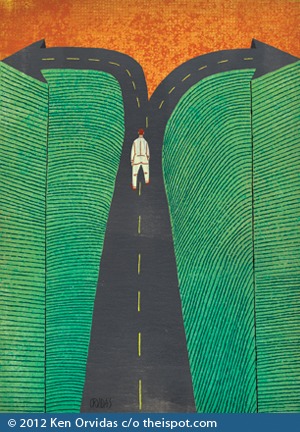
Rapid advances in biotechnology and genetics over the past 50 years have transformed medicine, agriculture, and industry. But these same developments have occurred at such a pace that the scientific community is only just beginning to address some of the new practical and ethical issues that have arisen. For instance, some research conducted in the service of humanity also carries inherent risks related to national security.^^1^^ This so-called dual-use research, with its potential to be used for both beneficial and detrimental purposes, raises thorny questions for investigators, funding agencies, and science journals worldwide.

## New Policy Meets New Research

Dual-use research is the subject of a new policy issued by the White House on 29 March 2012.^^2^^ The policy was issued in the midst of an ongoing international debate surrounding two articles submitted to *Nature* and *Science*. Each article documents successful attempts at making the deadly avian influenza (H5N1) virus more easily transmissible between mammals—and hence, potentially, a better biological weapon—setting off a firestorm within the scientific community.

Both studies used ferrets as a model to demonstrate that a genetically modified H5N1 virus could become readily transmissible between mammals, although it remains unclear how this virus would affect humans. In addition to concerns over how would-be terrorists might use the research, grave concerns exist that an engineered H5N1 virus could escape from the laboratory. According to the World Health Organization (WHO), H5N1 kills 60% of the people it infects,^^3^^ although this figure is much debated since it does not include people who may get infected but do not visit a doctor.^^4^^ In nature, the virus transmits rarely from birds to people (infecting only those in close proximity to birds, such as poultry workers) and even more rarely from person to person.

The two papers were sent to the National Science Advisory Board for Biosecurity (NSABB), established by the U.S. government after the anthrax attacks of 2001 to provide advice, guidance, and leadership on national security matters related to dual-use research.^^5^^ Unlike most scientists, NSABB members have received “Secret” security clearance and thus are equipped to assess national security risks of research. After deliberating amongst themselves and talking with both papers’ lead authors (Ron Fouchier of Erasmus Medical Center in the Netherlands and Yoshihiro Kawaoka of the University of Wisconsin–Madison), in December 2011 all 23 NSABB members voted unanimously to recommend the two journals redact key parts of the manuscripts, allowing the sensitive portions to be made available to researchers only on a need-to-know basis. Although under no legal obligation to do so, the journals and researchers agreed, although *Science* stipulated that the U.S. government would need to provide a “written, transparent plan” for making the redacted information available for “all those responsible scientists who request it” as part of their work.^^6^^

On the heels of the NSABB decision, 39 influenza researchers including Fouchier and Kawaoka voluntarily agreed to a 60-day moratorium on H5N1 research,^^7^^ which was later informally extended.^^3^^ NSABB chairman Paul Keim, a biology professor at Northern Arizona University, called this “an Asilomar moment” in scientific history, referring to the 1975 gathering of scientists at the California conference site to draw up guidelines for safely advancing then-nascent recombinant DNA technology—a meeting widely hailed as a model for scientific self-regulation.^^8^^

With the research moratorium in place and the papers delayed, scientists and public-health officials convened several international meetings to debate the dilemma. Should certain research on dangerous pathogens be government-classified to restrict access, as some nuclear research is, or can the scientists regulate their own work? Should this and other research with the potential for dual use even be conducted at all? Once a study is completed, can research results ever be fully contained? And what is the best way to get the broader scientific community thinking about the dual-use potential of their own research?

During the months after the NSABB’s initial decision, accusations and heated words flew across the Internet, in the media, and at conferences. “The New York Academy meeting I attended on this^^9^^ was as hostile a meeting as I can ever recall,” says Ron Atlas, a biology professor at the University of Louisville and coauthor of a seminal 2004 National Research Council document on dual-use research.^^1^^ “The panel was split into those who absolutely felt the [H5N1] research should go ahead and those who felt we needed to put it all back in the box.”

**Scope of the Federal Policy on Dual-Use Research**^2^

This policy calls for the regular review of research funded or conducted by the government with regards to its potential for dual use. Reviewers will focus onresearch involving one or more of the agents in List A that intentionally or unintentionally produces or could produce one or more of the effects listed in List B.

- Pathogens and toxins
  Avian influenza (H5N1) virus
  Bacillus anthracis
  Botulinum neurotoxin
  Burkholderia mallei
  Burkholderia pseudomallei
  Ebola virus
  Foot-and-mouth disease virus
  Francisella tularensis
  Marburg virus
  Reconstructed 1918 influenza virus
  Rinderpest virus
  Toxin-producing strains off *Clostridium botulinum*
  Variola major virus
  Variola minor virus
  Yersinia pestis
- Effects
  Enhances the harmful consequences of the agent or toxin.
  Disrupts immunity or the effectiveness of an immunization against the agent or toxin without clinical or agricultural justification.
  Confers to the agent or toxin resistance to clinically or agriculturally useful prophylactic or therapeutic interventions against that agent or toxin or facilitates their ability to evade detection methodologies.
  Increases the stability, transmissibility, or the ability to disseminate the agent or toxin.
  Alters the host range or tropism of the agent or toxin.
  Enhances the susceptibility of a host population to the agent or toxin.
  Generates or reconstitutes an eradicated or extinct agent or toxin from the list above.

Then the situation took an unexpected turn. A February 2012 meeting convened at the WHO headquarters in Geneva culminated in a letter signed by 22 scientists and public-health officials from 11 nations calling for full publication of the H5N1 papers—in contrast to the NSABB’s recommendation.^^10^^ A few days later, the United States asked the NSABB to reconsider revised versions of the manuscripts with additional information about the transmissibility of the engineered viruses from both studies. In a surprise move, the NSABB reversed course, recommending full publication. The board’s consensus was that the research was not as dangerous as it initially seemed. However, that was not the only factor in the switch.

“The truth is that they found out they couldn’t make redaction work in the sense that there wasn’t a good international mechanism for forwarding the details to people who needed to know, and no agency was willing to take it on,” says NSABB member Ken Berns, a professor of molecular genetics at the University of Florida. Classification and control of research also has potential to undermine international cooperation under the Biological and Toxin Weapons Convention of 1975, a multilateral disarmament treaty prohibiting the use, possession, and production of biological weapons.

The *Nature* article was published online 2 May 2012.^^11^^ At press time, the *Science* article had not been published.

## Dual-Use Research in Many Disciplines

As defined in the 2004 National Research Council report colloquially called the Fink Report,^^1^^ dual-use research is that which could be misapplied to cause substantial damage to human health, agriculture, the environment, the economy, or national security. A subset of dual-use research that is deemed by the NSABB to be especially harmful if misapplied is known as “dual-use research of concern” (DURC).^^12^^

Dual-use research can occur in many academic disciplines. During the advent of atomic physics, Leó Szilárd, the discoverer of the nuclear chain reaction, realized its potential for mass destruction.^^13^^ He discussed with other nuclear scientists whether they should self-regulate their work and keep results secret to avoid nuclear reactions being weaponized. Indeed, discoveries subsequently published by other physicists eventually led to the creation of the atomic bomb; today, most U.S. nuclear weapons research is classified.^^14^^ Dual-use research may also include encryption and cryptography, psychological research that could be used to develop mind-control techniques,^^15^^ engineering research including weaknesses of the electric power grid or building design flaws, and research on public-health vulnerabilities.

One of the most contentious papers in dual-use history is a 2005 study that showed how a mere 4 g of botulinum toxin dispersed at one dairy plant could kill 400,000 people.^^16^^ The authors’ intent was to show how to protect the U.S. milk supply, but the paper came under fire by the government because the information could easily be countermined for malicious intent. The Department of Health and Human Services asked the *Proceedings of the National Academy of Sciences* not to publish the paper,^^17^^ but the journal proceeded with publication, opining in an editorial not only that the public should be made aware of the danger but that free and open scientific inquiry ultimately makes the public safer.^^18^^

The potential for dual-use research in the environmental health sciences is less clear-cut, but it does exist. For instance, in 1943, botany student Arthur Galston published his thesis on chemicals that hasten the development of flowering plants. After military researchers read the thesis, they used Galston’s findings to develop the defoliant Agent Orange, a chemical whose use in the Vietnam War has caused human health problems ever since.^^19^^ The thesis would not likely have been flagged as dual-use research because Galston did not address military tactics. But the example illustrates how seemingly innocuous research can lead to unintended consequences.

David Resnik, a bioethicist with the National Institute of Environmental Health Sciences (NIEHS), says most environmental-health research that could cause harm would not do so on the scale or the scope that could be expected with something like dangerous pathogens. Nevertheless, the possibility does exist, and training is conducted across the National Institutes of Health (NIH), including the NIEHS, to alert researchers to dual-use potential.

Jonathan Suk, a social scientist at the European Centre for Disease Prevention and Control, and his colleagues argue that more attention should be paid to low-tech threats, such as direct contamination of food and water, given the much lower technical hurdles involved compared with obtaining, engineering, or weaponizing a dangerous pathogen. They point out that the only known case where a terrorist group employed a biological agent in the United States involved a low-tech attack: In 1984 the Rajneeshee cult contaminated salad bars in The Dalles, Oregon, with *Salmonella*, which caused 751 illnesses but no deaths. On the other hand, the Japanese cult Aum Shinrikyo was unsuccessful in its attempts to obtain and disperse anthrax and botulinum toxins, and it is believed that al-Qaeda attempted to obtain the means to create a biological weapon in the early 2000s but failed to do so.^^20^^

## Self-Regulation versus Government Regulation

The newly released federal policy on dual-use research aims to catch research at the proposal stage, because dealing with a completed manuscript once it is submitted to a journal causes numerous practical challenges.^^21^^ For instance, journal editors may be unable to adequately assess the biosecurity risks of research, and once a manuscript is written it is virtually impossible to control its spread worldwide.

The policy applies to all extramural and intramural research funded by the government but not to research funded by other sources. Under the policy, all research-funding government agencies must review currently proposed and ongoing research projects to identify those that involve any of 15 high-concern pathogens and toxins (see box). For projects identified as such, researchers are required to develop “risk mitigation plans.” If they are unable to develop an acceptable plan, the policy states that “Federal departments and agencies will determine whether it is appropriate to: (a) request voluntary redaction of the research publications or communications; (b) classify the research . . . [or] (c) not provide or terminate research funding.”^^2^^

Berns is pleased that in issuing the policy the White House finally acted on a recommendation the committee made six years ago. “There was a lot of concern [among NSABB members] over waiting until work gets sent for publication to think about its dual-use implications,” he says. “This way, before the whole [study] gets started, people can think about what it could mean, what the issues might be, and both the researchers and funders can act accordingly.”

Some argue that researchers may not always willingly put national security interests above their own career advancement.^^13^^^^,^^^^22^^ “Meaningful self-regulation has not been forthcoming from within the science community,” says Rutgers University microbiologist Richard Ebright, an outspoken critic of the two H5N1 studies, who believes they should never have been conducted.

Ebright prefers a proactive approach on the part of the government. “Oversight needs to come from the public and policy makers,” he says. “Pathogens researchers have made it clear: Left to their own devices, they will disregard the public interest.”

But researchers may not always be capable of assessing the dangers of their research. Michael Selgelid, director of the Centre for Human Bioethics at Monash University in Australia, warned against this in a 2009 *Bulletin of the World Health Organization* article, writing, “Because scientists generally lack training in security studies, they may lack the expertise required for assessment of the security risks of publication in any given case.”^^13^^

One example of this lack of expertise is an article published in the *Journal of Virology* documenting the creation of a potent mousepox strain that killed mice normally resistant to the virus.^^23^^ Although not dangerous to humans, the same technique could potentially be used to create a more virulent smallpox strain, yet neither the researchers nor the journal editors had access to government-classified information about smallpox proliferation. With access to such information, the researchers potentially could have assessed the dual-use possibilities of their research before conducting it and acted accordingly.

One perceived challenge of increased oversight of dual-use research includes concerns that such oversight could soak up time, money, and personnel. However, two separate surveys by NIH investigators suggest otherwise. Molly S. Stitt-Fischer and colleagues reviewed 3,444 annual research progress summaries submitted to NIH in 2009 and flagged just 2.9% in an initial screening as having dual-use potential.^^24^^ Megan C. Morgan and colleagues reviewed 734 research registration documents submitted to the NIH Institutional Biosafety Committee (IBC) between 2004 and 2008. In an initial screening they flagged 1.6% as potentially being in the more restrictive DURC category, but after a more detailed review they determined that none of the studies’ research data or products could be readily utilized to cause public harm.^^25^^

“Overseeing just [a minuscule fraction] of research projects could not possibly ‘bring science to a stop,’ as some have argued,” says Ebright. Indeed, he argues that failing to monitor these projects actually poses the greater risk for science. Without oversight, he says, those few DURC projects put the vast majority of studies at significant risk. “When the first major lab accident with an engineered pathogen occurs, there will be a draconian reaction from the public and policy makers,” he says. “That reaction will not be focused on the very small group that needs oversight but on biomedical research as a whole.”

## Putting Awareness into Action

But some investigators are concerned at the prospect of increased government surveillance of research in the name of national security. “I think most scientists want to publish their research without worrying about red tape or political controversy,” says Resnik. “Most journal editors don’t want to deal with outside interference from the government or any other organization.”

Resnik and colleagues at the NIEHS conducted a random survey of 400 life-sciences journals to determine how many had dual-use policies in place. Of the 155 that responded, only 7.7% had a written policy for how to deal with dual-use manuscripts that may cross their desk, with 5.8% having reviewed such a study during the previous five years. In written responses to the survey, one editor reported having reviewed what turned out to be dual-use manuscripts but had never heard the term “dual use.” Some responded negatively to the idea of a dual-use policy. For instance, one editor appeared to believe it would involve employing two different methods of peer review.^^26^^

*EHP* began actively evaluating papers for their dual-use potential in 2009 and in February 2010 added a statement to its Instructions to Authors^^27^^ that additional expert advice would be sought if the editors had concerns about a manuscript. “The editors of *EHP* strongly support the unrestricted communication of research findings, but the journal also has a responsibility not to publish work that could be readily used to cause public harm,” explains *EHP* science editor Jane Schroeder. “To date we have not received any such papers, which is not surprising given that most research that would be considered ‘dual use’ is outside *EHP*’s scope. However, we will continue to monitor papers submitted to *EHP*.”

If researchers consider dual-use potential in their work from the outset, they are more likely to be in a position to evaluate benefits and risks of a particular project and to mitigate any potential risks before a project begins, and less likely to meet controversy when such work reaches publication stage. “Scientists really ought to be alert to the question of whether or not what they’re doing could be misused in a way that could be harmful,” says Berns.

The bones of a system to review proposals for dual-use potential exists with university-level IBCs, but the true mission of these committees is to review recombinant DNA technology and laboratory safety. “My sense is that those groups don’t think about dual use, just biosafety, and it’s a burden to put on a different hat,” says Atlas.

An informal poll of various institutions conducted in the course of writing this article suggests it’s a mixed bag as to whether a given university’s IBC has any dual-use policy, although this was one of the key recommendations in the Fink Report.^^1^^ “That idea has not been widely accepted in the scientific community,” Atlas says.

When scientists met in Asilomar in the 1970s, they applied the precautionary principle in working out a consensus statement on how to regulate their own work on recombinant DNA technology.^^21^^ The present “Asilomar moment” has the potential to work itself out as successfully as the early use of that biotechnology did.^^8^^ But as an issue of global import, international cooperation will certainly be necessary to ensure public safety as well as the march of scientific progress.

“Even to the extent that the U.S. classifies research, they can only classify U.S. research; they can’t classify foreign research,” says Atlas. “The ultimate solution lies with voluntary action of the life-sciences community. That’s not to say there should not be government advice, such as the NSABB, but mandatory controls won’t work when you’re dealing with a global enterprise. It remains to be seen how you make this a globally accepted policy.”

## References

[1] National Research Council (2004). Biotechnology Research in an Age of Terrorism.. http://www.nap.edu/catalog.php?record_id=10827.

[2] OBA. United States Government Policy for Oversight of Life Sciences Dual Use Research of Concern [website]. Bethesda, MD:Office of Biotechnology Activities, Office of Science Policy, National Institutes of Health. Available: http://oba.od.nih.gov/oba/biosecurity/PDF/United_States_Government_Policy_for_Oversight_of_DURC_FINAL_version_032812.pdf [accessed 7 May 2012].

[3] WHO. Public Health, Influenza Experts Agree H5N1 Research Critical, but Extend Delay [press release]. Geneva, Switzerland:World Health Organization (17 Feb 2012). Available: http://www.who.int/mediacentre/news/releases/2012/h5n1_research_20120217/en/index.html [accessed 7 May 2012].

[4] Racaniello VR (2012). Science should be in the public domain.. mBio.

[5] OSP (2012). About NSABB [website].. http://oba.od.nih.gov/biosecurity/about_nsabb.html.

[6] AAAS. Statement by Science Editor-in-Chief Dr. Bruce Alberts Regarding Publication of H5N1 Avian Influenza Research [press release]. Washington, DC:American Association for the Advancement of Science (20 Dec 2011). Available: http://www.aaas.org/news/releases/2011/media/1220herfst_statement.pdf [accessed 7 May 2012].

[7] Fouchier RAM (2012). Pause on avian flu transmission research.. Science.

[8] Berg P. Asilomar and Recombinant DNA [website]. Stockholm, Sweden:Nobel Media (26 Aug 2004). Available: http://www.nobelprize.org/nobel_prizes/chemistry/laureates/1980/berg-article.html [accessed 7 May 2012].

[9] Academy eBriefings: Dual Use Research: H5N1 Influenza Virus and Beyond. [website]. New York, NY:The New York Academy of Sciences (updated 24 Feb 2012). Available: http://www.nyas.org/Publications/EBriefings/Detail.aspx?cid=4cd6eec7-7bbf-4be8-b9d3-b458937c043d [accessed 7 May 2012].

[10] Cohen J. WHO group: H5N1 papers should be published in full. Science 335 6071 899 900 2012 http://dx.doi.org/10.1126/science.335.6071.899 22362977 10.1126/science.335.6071.899

[11] Imai M Experimental adaptation of an influenza H5 HA confers respiratory droplet transmission to a reassortant H5 HA/H1N1 virus in ferrets. Nature 2012); doi: [online 2 May 2012] 10.1038/nature1083 PMC3388103 22722205

[12] NSABB. Proposed Framework for the Oversight of Dual Use Life Sciences Research: Strategies for Minimizing the Potential Misuse of Research Information. A Report of the National Science Advisory Board for Biosecurity (NSABB). Washington, DC:National Science Advisory Board for Biosecurity (Jun 2007). Available: http://oba.od.nih.gov/biosecurity/pdf/Framework%20for%20transmittal%200807_Sept07.pdf [accessed 7 May 2012].

[13] Selgelid MJ (2009). Governance of dual-use research: an ethical dilemma.. Bull WHO.

[14] Office of Classification Mission and Function [website]. Washington, DC: Office of Health, Safety, and Security, Department of Energy. Available: http://www.hss.doe.gov/classification/ [accessed 7 May 2012].

[15] Moreno JD (2003). Neuroethics: an agenda for neuroscience and society.. Nat Reviews Neurosci.

[16] Wein LM, Liu Y (2005). Analyzing a bioterror attack on the food supply: the case of botulinum toxin in milk.. Proc Natl Acad Sci USA.

[17] Re: The article “Analyzing a Bioterror Attack on the Food Supply: The Case of Botulinum Toxin in Milk” by Lawrence Wein and Yifan Liu. Letter from Stewart Simonson to Bruce Alberts. 27 May 2005. Available: http://www.fas.org/sgp/bush/hhs052705.pdf [accessed 7 May 2012]. 10.1073/pnas.0408526102 PMC1161865 15985558

[18] Alberts B. Modeling attacks on the food supply. Proc Natl Acad Sci USA 102 (28 9737 9738 2005 http://dx.doi.org/10.1073/pnas.0504944102 15985557 10.1073/pnas.0504944102 PMC1175018

[19] Lilienfeld DE, Gallo MA (1989). 2,4-D, 2,4,5-T, and 2,3,7,8-TCDD: an overview.. Epidemiol Rev.

[20] Suk JE (2011). Dual-use research and technological diffusion: reconsidering the bioterrorism threat spectrum.. PLoS Pathog.

[21] Shea DA. Balancing Scientific Publication and National Security Concerns: Issues for Congress. CRS Report for Congress. Washington, DC:Congressional Research Service (updated 2 Feb 2006). Available: http://www.fas.org/sgp/crs/secrecy/RL31695.pdf [accessed 7 May 2012].

[22] Resnik DB (2010). Can scientists regulate the publication of dual use research?. Stud Ethics Law Technol.

[23] Jackson RJ (2001). Expression of mouse interleukin-4 by a recombinant ectromelia virus suppresses cytolytic lymphocyte responses and overcomes genetic resistance to mousepox.. J Virol.

[24] Stitt-Fischer MS. The National Institutes of Health Dual Use Screening Program: A Proposed Quality Control Mode [speech]. Presented at: Annual Biological Safety Association Conference, Denver, CO, 5 Oct 2010. Available: http://www.absaconference.org/pdf53/Session11-Stitt-Fischer.pdf [accessed 7 May 2012].

[25] Morgan MC. Evaluation of a First-Tier Screening Program for Dual Use Research of Concern [speech]. Presented at: Annual Biological Safety Association Conference, Denver, CO, 5 Oct 2010. Available: http://www.absaconference.org/pdf53/Session11-Morgan.pdf [accessed 7 May 2012].

[26] Resnik DB (2011). Dual-use review policies of biomedical research journals.. Biosecur Bioterror.

[27] EHP. Instructions to Authors [website]. Research Triangle Park, NC:Environmental Health Perspectives, National Institute of Environmental Health Sciences (2012). Available: http://ehp03.niehs.nih.gov/static/instructions.action [accessed 7 May 2012].

